# Trends in hospitalizations for diagnosed congenital cytomegalovirus in infants and children in Australia

**DOI:** 10.1186/1471-2431-9-7

**Published:** 2009-01-26

**Authors:** Holly Seale, Robert Booy, C Raina MacIntyre

**Affiliations:** 1 School of Public Health and Community Medicine, Faculty of Medicine, University of New South Wales, Kensington, New South Wales, Australia; 2 National Centre for Immunization Research and Surveillance of Vaccine Preventable Diseases, Children's Hospital at Westmead, The University of Sydney, Sydney, New South Wales, Australia

## Abstract

**Background:**

Cytomegalovirus (CMV) is responsible for a wide range of diseases in neonates, and has been recognized as a major cause of congenital defects in developed countries for many years. More children suffer serious disabilities caused by congenital CMV than by several better-known childhood maladies. Insight into the epidemiology of congenital CMV disease is needed for the assessment of preventive strategies.

**Methods:**

Using data from the National Hospital Morbidity Database (NHMD) complied by the Australian Institute of Health and Welfare (AIHW), we examined the rates of hospital admissions for children diagnosed with congenital cytomegalovirus (CMV) by year, sex, age group and length of stay.

**Results:**

Over an 8-year period (1993–2001), there were 1314 admissions for the congenital form of CMV disease. Of these admissions, 25% were principally hospitalized because of congenital CMV. The average annual rate of admissions in children aged 0–4, 5–9 and 10–14 years was 9.40, 2.40 and 0.85 per 100,000 Australian population respectively.

**Conclusion:**

Compared with many other congenital illnesses, which are now vaccine preventable, the burden of congenital CMV is comparatively high. A vaccination program would be justifiable should a vaccine become available.

## Background

An estimated 20 000 – 40 000 infants are born each year with congenital cytomegalovirus (CMV) infection in the United States alone. About 100–200 of these infants will die as a consequence of symptomatic infection and a further 4000–8000 will develop permanent neurological complications that often lead to permanent disabilities [1]. Higher estimates of the burden of congenital CMV in terms of death and disability have also been published [2,3].

The number of US infants experiencing symptoms from congenital CMV infections exceeds the number of newborns affected by other, better-known childhood diseases and syndromes, such as congenital rubella, fetal alcohol syndrome, down's syndrome and neural tube defect [4,5]. While this large difference in occurrence is partly due to improvements in the screening and prevention of these other conditions, congenital CMV infections have occurred at these high rates for decades. Before the development and availability of the rubella vaccine, it was noted that the year-in and year-out toll of CMV infection exceeded that of rubella, but the impact of rubella was more dramatic because it was epidemic in nature [6].

Most children with asymptomatic congenital CMV infection develop normally without any permanent cognitive, perceptual or motor deficits [4,6]. However, for some children who are asymptomatic at birth there is the risk that they may develop CMV-related symptoms, such as visual impairment [5,7-11]. Some researchers have suggested that asymptomatic congenital CMV infection can impair intellectual development and neurophysiologic performance [12-15]. Although visual impairment is less common in asymptomatic infants compared to those who are symptomatic at birth; chorioretinitis, optic atrophy, strabismus and pigmentary retinopathy do occur in 1.2%–3.9% of cases [11,16,17]. Each year, an estimated 2,600 children develop chorioretinitis or optic atrophy in the United States from asymptomatic congenital CMV infections, with approximately 15% having a visual acuity of worse than 20/50 in one eye [11].

For infants with symptomatic congenital CMV infection the outcome is poor [18]. Around 10%–15% will experience symptoms at birth [7]. Jaundice, hepatosplenomegaly and petechiae are the most frequently reported signs of congenital CMV infection in the neonate, being present in up to two thirds of overtly infected infants [5,19,20]. Neurological signs of congenital CMV infection include microcephaly, mental retardation (IQ<70), motor abnormalities, sensorineural hearing loss (bilateral and unilateral), chorioretinitis and other visual defects and seizures [17,21-24]. Sensorineural hearing loss (SNHL) is the most common symptom of CMV infection among the 10–15% of children with symptoms of infections at birth [25,26]. These cases account for at least one-third of all SNHL in children [27]. While there are multiple causes of hearing loss, CMV is the only significant viral cause of hearing loss, since the introduction of vaccination for measles, mumps and rubella, has made hearing loss from those causes extremely rare [28].

Children with congenital CMV-induced sequelae have a wide range of special needs, and they often require extensive interventions from health care providers, such as cochlear implantation, speech therapy, neurodevelopmental assessments, and, in severe cases, lifelong custodial care.

As a vaccine for CMV may one day be available, understanding the pre-vaccination burden of CMV disease is useful for planning future prevention programs. To examine the epidemiology of congenital CMV in Australia, a retrospective review of hospital admissions was undertaken. The objectives were to analyze the rate, temporal trends and patient mortality associated with these hospital admissions.

## Methods

A retrospective analysis of hospital discharge records from 1993 to 2001 was conducted. The data were drawn from the National Hospital Morbidity Database (NHMD) complied by the Australian Institute of Health and Welfare (AIHW) from data supplied by the State and Territory health authorities [29]. The NHMD is a collection of electronic confidentialised summary records for admitted patients dying/discharged from public and private acute and psychiatric hospitals as well as private free-standing hospital facilities in Australia. The National Health Data Dictionary definitions form the bases of the databases, ensuring a high standard of data comparability. Hospitalizations were chosen because they were more accessible in the database and less subject to interpretation than outpatient cases of CMV disease. Ethical clearance for the overall study was sought and obtained from the Western Sydney Area Health Service.

Data was extracted on patients admitted into hospital with either a principal or non-principal diagnosis of congenital CMV. Patients with a non-principal diagnosis were also included to get a more complete picture of the burden of disease attributable to CMV. All data used had been previously de-identified by the AIHW. The data were analyzed by financial year based on the date of hospital discharge. All patients discharged between July 1, 1993 and June 30, 2001 was included. Hospital discharge diagnoses and procedures were coded in accordance with the International Classification of Diseases, 9th and 10th Revision (ICD9 and ICD10) Australian Modification. Any admission with a specific congenital CMV ICD 9 code: 777.1 for the period 1993–1998, or ICD-10: P35.1 for the period 1999–2001, was included.

The age groups were 0–4, 5–9 and 10–14 years. A record is included for each separation, not for each patient, so patients who separated more than once in the year have more than one record in the database. The variables that were extracted for analysis included: sex, age, length of stay, separation mode (which includes death as an outcome of hospitalization) and diagnosis. Data from all States and Territories were available for analysis.

In addition to examining the geographic location of the admission, the Rural, Remote and Metropolitan Areas (RRMA) classification was also examined. This classification was developed in 1995 to differentiate between regions. The RRMA has seven categories, which collapse into three zones. The metropolitan zone includes the categories capital cities and other metropolitan centers; the rural zone includes large rural centers, small rural centers and other rural areas, while the remote zone includes remote centers and other remote areas. RRMA values are based on the population density of the Statistical Local Area (SLA).

Rates were calculated using Australian Bureau of Statistics (ABS) mid-year estimated resident information for children and adolescents aged 0–14 years [30]. Rates are presented as annual rates or average annual rates per 100 000 population (0–14 years) or population in age, sex as appropriate. Average annual rates were calculated by dividing the total number of cases for the period of investigation by the sum of each year's population for the same period. For hospitalization data, the midyear population estimate for the first half of the financial year was used as the denominator.

## Results

**Figure 1:**
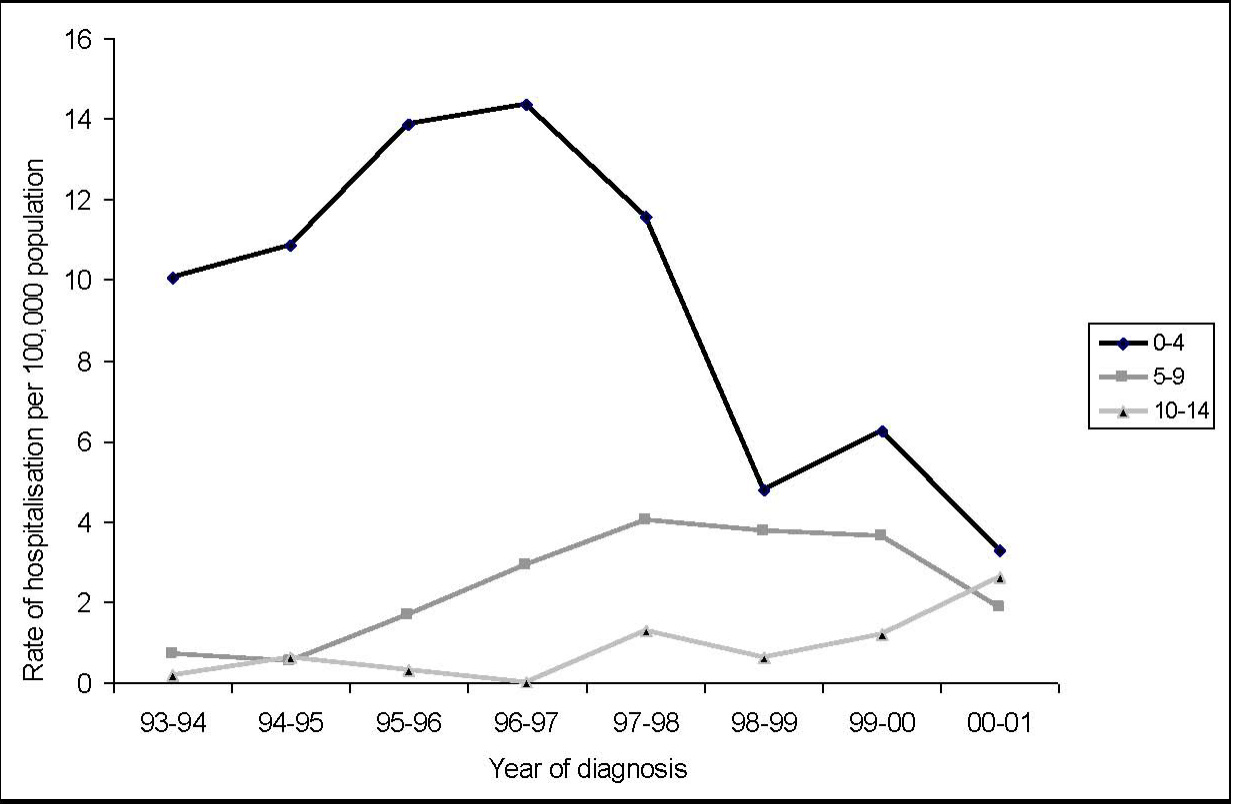
**Congenital CMV age-specific hospital rates by age group (aged ≤ 14 years) and year of diagnosis, Australia, July 1993 to June 2001**.

From July 1993 to June 2001, there were 1314 admissions for children (aged ≤ 14 years) diagnosed with congenital CMV disease, giving an average annual rate of 4.2 cases per 100,000 children ≤ 14 years. The rate peaked in 1996–1997 at 5.7 cases per 100,000 and then declined to 2.6 cases per 100,000 in 2000–2001 (Figure 1).

The average age specific annual rate of admission for children aged 0–4, 5–9 and 10–14 years was 9.40, 2.40 and 0.85 per 100,000 respectively between 1993 and 2001 (Table 1). The overall male to female ratio was 1.3:1 admissions. The average annual rate of male admissions for the eight years (4.0 cases per 100,000 male population aged ≤ 14 years) exceeded the female rate (3.1 cases per 100,000 female population aged ≤ 14 years). Over the eight year period, 39,818 bed days (average 4977 days per year) were recorded. The mean length of stay was 30 days, whilst the median length of stay was 7 days (range: 1 to 86 days), and 34% of hospitalizations were for 48 hours or less.

**Table 1:** CMV hospitalisations by age group (0–14 years), Australia, July 1993–June 2001

**Age group (years)**	**Hospitalisations July 1993–June 2001**		LOS* per admission (days)	**Deaths (number)**
	No. (PD ^†^)	Rate‡	Median	No. (PD ^†^)

0–4	971 (150)	9.40	15	23 (8)
5–9	253 (137)	2.40	2	0
10–14	90 (42)	0.85	5	0

Total	1314 (329)	3.2	7	23

†PD = principal diagnosis

*LOS = length of stay in hospital

‡ Average annual age-specific rate per 100,000 population

**Figure 2:**
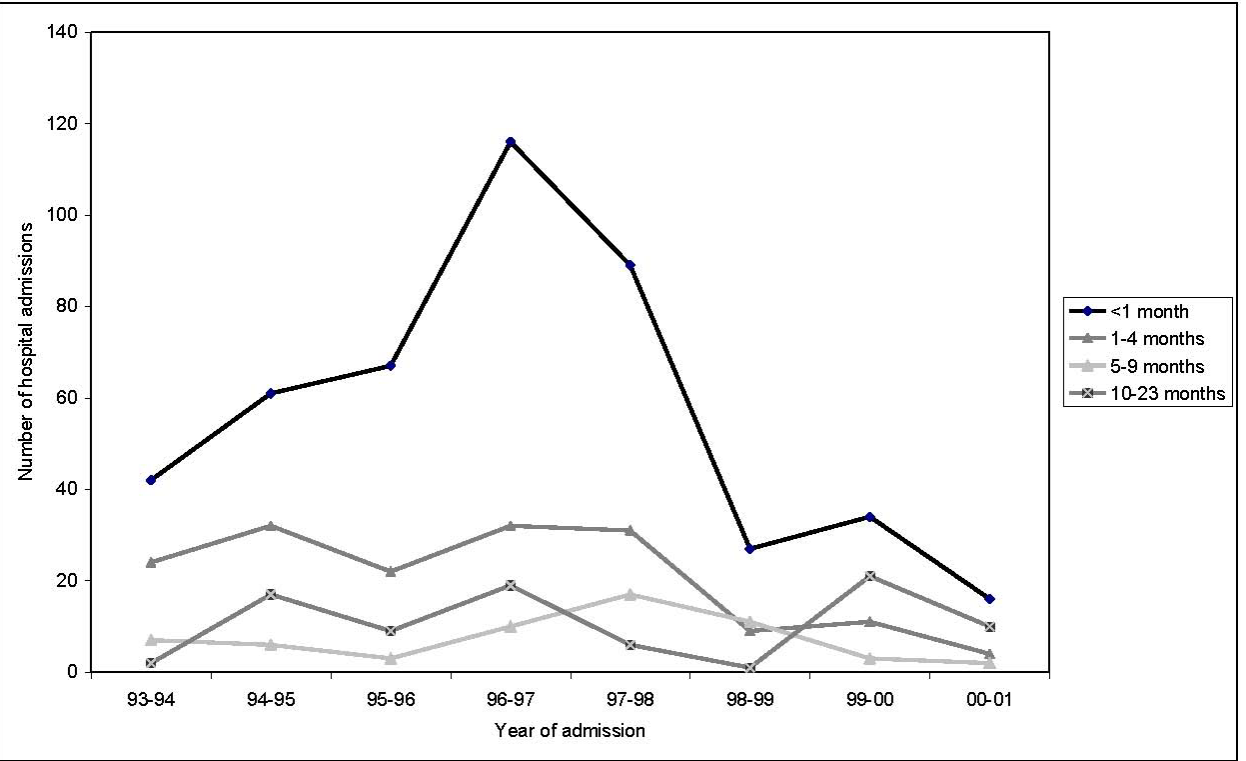
**Number of hospital admissions for congenital CMV by age group (aged ≤ 23 months) and year of diagnosis, Australia, July 1993 to June 2001**.

Out of the 1314 hospital admissions, 57.9% were recorded for a patient aged ≤ 23 months. The average annual admission rate for infants aged ≤ 23 was 62.3 per 100,000 Table 2). Over the eight year period, admissions peaked for children aged <1 month in 1996–1997, after which time admissions declined again. For the other age groups, there were minor peaks registered for each group, but they were not as dramatic as they were for the youngest age category (Figure 2). In patients aged less than one month, the average annual admission rate was 443 per 100,000 compared to only 9.3 per 100,000 for children aged between 10 and 23 months. Females accounted for 50% of all admissions for this age bracket. Children aged four months and over had a significantly lower risk of being hospitalized with CMV compared with children aged less than one month, and this risk decreased with increasing age. All fatal cases of CMV were in children aged less than 12 months (23 cases over 8 years).

**Table 2:** CMV hospitalisations by age group (<1–23 months), Australia, July 1993 – June 2001

**Age Group (months)**	**Hospitalisations July 1993–June 2001**		**Average annual rate per 100,000**
	**No**.	**%**	
<1	452	59.4	443.7
1–4	165	21.7	162.0
5–9	59	7.8	57.9
10–23	85	11.2	9.3

TOTAL	761	100	62.3

The highest average annual rate of admissions was recorded for the Northern Territory (5.68 per 100,000 NT population), followed by New South Wales (4.40 per 100,000 NSW population) and the South Australia (3.49 per 100,000 SA population). The highest annual rate of admission was 20.6 per 100,000, recorded in the Northern Territory in 1996–1997. This was followed by 10.9 per 100,000 in the Australian Capital Territory in 1997–1998 and 8.33 per 100,000 in South Australia in 1996–1997. Information on the RRMA was available from 1995 onwards for the admissions with congenital CMV. From the database, we found that 71.7% (741/1033) of admissions were classified as being in a metropolitan area, 18.2% (188/1033) were in rural areas, 4.6% (48/1033) were in remote areas and 5% (56/1033) were not coded. Of the remote hospitalizations, 54% (26/48) were from the Northern Territory, 21% (10/48) were from South Australia and 12.5% (6/48) from both Queensland and Western Australia.

## Discussion

The rate of admissions for congenital CMV was found to be highest in infants aged less than five years, amongst whom the average annual hospitalization rate was 9.40 cases per 100,000 population. This group accounted for the highest percentage of hospitalizations (74%) and all deaths. This probably reflects the fact that the likelihood of diagnosis is highest in this age group.

One of the aims of this paper was to assess the burden that congenital CMV has on the health system. From the NHMD, it was established that over the eight years, congenital CMV was the principal cause of admission in 329 cases, and was diagnosed in a further 985 admissions. It is well recognized that children with symptomatic congenital CMV require complex medical and surgical care as a result of the consequences of this infection. Previous studies have estimated that the disease burden associated with congenital CMV disease in the early 1990's, cost the US healthcare system approximately $US1.9 billion annually, and a cost per affected child of over $US300, 0000 [31]. These costs reflect the lifelong disability associated with symptomatic infection, since patients often require long-term residential care and extensive medical intervention [32]. To the best of our knowledge, the costs associated with congenital CMV disease have not been calculated in Australia. From the information obtained from the NHMD, it is possible to calculate the cost per case associated with a hospital visit. This performance indicator is a measure of the average cost of providing care for an admitted patient, adjusted for the relative complexity of the patient's condition and hospital services provided. Although this calculation is not specific to patients with CMV disease, it does provide a rough estimate of the costs attributable to the disease. For example, in 2000–2001, the cost per case mix-adjusted separation was $2,834. As there were 104 admissions, the estimated cost attributable to congenital CMV for that year would be $294 736. Further studies would need to be conducted to ascertain financial costs associated with this disease.

The second aim was to establish up-to-date estimates of the burden of congenital CMV in Australia. Due to the nature of the hospital database used in this study, it was impossible to ascertain a true estimate of the burden, as we could not separate children who had recurrent admissions. There are however, a number of previous studies which have tried to document the rate of congenital CMV in Australia. One of the first studies [33] examined 47,320 consecutive live births at an obstetric hospital, and found that one in every 4000 child born had evidence of neonatal cytomegalic inclusion disease (CID). From these results, the authors suggested that a very low rate of disease existed in Australia for congenital CMV. However, this study was limited to only including children who were symptomatic at birth and excluded any asymptomatic children, therefore underestimating the burden of the disease. Given that 80–90% of infected children are asymptomatic at birth, extrapolation of the reported incidence from the study provided an estimate of total incidence closer to 0.2–0.3% [34]. In a later study, Sfameni et al., found that firstly 71% of the pregnant women screened in the study had antibodies to CMV at their first antenatal visit and secondly that primary CMV infection occurred in five of 338 (1.5%) initially seronegative women whose sera were retested at the end of their pregnancies. On the basis of these observations the authors estimated that congenital CMV infection due to primary maternal infection occurred in up to 2 in 1,000 infants in the community [35]. However, this calculation also does not account for all illness associated with CMV. In 2005, Munro et al. used the primary infection rate described by Sfameni in 1986 (1.5%) and the number of births in Australian and estimated that approximately 1476 babies are born with congenital CMV in Australia and that 15% show symptoms at birth [36]. However, this is again still not a true reflection of the burden of CMV in Australia, as it only reflects only symptomatic cases at birth.

Worldwide, there are 15 surveillance units, which make up the International Network of Pediatric Surveillance Units (INoPSU) [37]. The network was established in 1998 to collectively conduct surveillance of uncommon conditions of childhood. Australia, Britain and Canada are the only countries which have undertaken active surveillance for congenital CMV. In Britain, during the 25 months of surveillance, 288 reports were received, of which 93 were confirmed cases, 69 suspected cases and 93 duplicate or error reports [38]. In Canada, surveillance, commenced in March 2005 and by December 31, 2007, there were 46 confirmed cases [39].

Although the surveillance systems used by the Network of Pediatric Surveillance Units have a definite advantage over more traditional passive reporting systems, complete case ascertainment is unlikely, especially amongst rural and Indigenous communities which are poorly served by pediatricians. For example, in Canada, the rate of infection is estimated to be around 1%, which would equate to approximately 300 cases per year from the Canadian birth cohort (assuming 10% of infants are symptomatic). This means approximately only 7% of cases are being reported the Canadian surveillance unit. The investigators suggest that this may be due to a number of factors including: that the neonatal symptoms may be subtle and not recognized as congenital CMV early enough in the neonatal period, or that the surveillance unit is only capturing a proportion of the diagnosed cases in the country [39]. The most accurate measurements of the infection rate will likely have to await a population-based surveillance to capture the full spectrum of congenital CMV in these countries. This has been successfully implemented for other congenital disease such as rubella.

Previous studies assessing the cost benefits of routine immunization for CMV, found that vaccination of healthy women aged 15–25 years is cost beneficial even in populations where CMV seroprevalence is high. In populations, such as Australia, where there is lower seroprevalence (57%), for every 100,000 women immunized, more than 24 cases of symptomatic congenital CMV infection at birth and a similar number of cases with late sequelae (mainly deafness) would be prevented yearly [31]. The Advisory Committee on Immunization Practices also suggested that if a vaccine program were implemented today, then the annualized present value of the Quality Adjusted Life Year (QALY) gained would be 70,000, with a US$4 billion dollar saving in health care costs [2]. These calculations were based on the assumption that the vaccine would be 100% efficacious and utilized by 100% of the target population (12-year-old boys and girls).

In contrast with the required vaccination rates needed for the elimination of measles (93%), mumps (93%) and rubella (92%), studies have shown that CMV requires a much lower rate of vaccination (60%). This is related to the fact that CMV has a low force of infection and a relatively low basic reproductive number (R0) of 2.5 [40]. R0 refers to the number of secondary infections caused by the introduction of a single infectious case into a completely susceptible population. The R0 for CMV is very similar to that of smallpox (Ro: 2.3–3.4) which required a critical vaccination proportion of 57% to 70% for eradication. Therefore, even if the efficacy of a candidate CMV vaccine was only 80–90% in preventing principal infection, the disease could be eradicated by immunizing 66–75% of the population [41]. However, just preventing principal infections may not be adequate enough. In highly immune populations secondary maternal infections, rather than primary attacks, may be the leading cause of intrauterine CMV infection [42]. Ahlfors et al. found that in their long-term study of maternal and congenital CMV infections that both primary and secondary infections were equally responsible for congenital CMV infection [6].

Our study has some limitations, mainly because of its reliance on hospital discharge data for which accuracy depends on the quality and consistency of coding by participating hospitals. As there were no unique identifying codes linking records for the same individual across databases, it was not possible to extract recurrent hospital visits for the same child. Therefore, data on some children were represented more than once for any given year. For hospitalizations where the CMV code was not the principal diagnosis, the CMV code will have been recorded as a co-morbidity (additional or secondary diagnosis), the relative importance of which cannot be gauged.

## Conclusion

These limitations notwithstanding, this nationwide look at trends in hospital use for children diagnosed with congenital CMV in Australia provides data that can be used to (1) justify the introduction of a vaccine when available, (2) define future policies in an era of competing health care priorities.

## Competing interests

The authors declare that they have no competing interests.

## Authors' contributions

HS participated in the design of the study and survey, performed the analysis and drafted the manuscript. RB participated in the design of the study and reviewed the manuscript. CRM helped perform the statistical analysis and drafted the manuscript. All authors have read and have approved the final manuscript.

## Pre-publication history

The pre-publication history for this paper can be accessed here:



## References

[B1] Ross SA, Boppana SB (2005). Congenital cytomegalovirus infection: outcome and diagnosis. Semin Pediatr Infect Dis.

[B2] Committee to Study Priorities for Vaccine Development (2000). Vaccines for the 21st Century: A Tool for Desionmaking.

[B3] Cannon M, Davis K (2005). Washing our hands of the congenital cytomegalovirus disease epidemic. BMC Public Health.

[B4] Demmler GJ (1991). Infectious Disease Society of America and Centers for Disease Control: Summary of a workshop on surveillance for congenital cytomegalovirus disease. Rev Infect Dis.

[B5] Fowler KB, Stagno S, Pass RF, Britt WJ, Boll TJ, Alford CA (1992). The outcome of congenital cytomegalovirus infection in relation to maternal antibody status. N Engl J Med.

[B6] Ahlfors K, Ivarsson SA, Harris S (1999). Report on a long-term study of maternal and congenital cytomegalovirus infection in Sweden. Review of prospective studies available in the literature. Scand J Infect Dis.

[B7] Stagno S, Whitley RJ (1985). Herpesvirus infection of pregnancy. Part I: Cytomegalovirus and Epstein-Barr Infections. N Engl J Med.

[B8] Alford CA, Stagno S, Pass RF, Britt WJ (1990). Congenital and perinatal cytomegalovirus infections. Rev Infect Dis.

[B9] Williamson WD, Percy AK, Yow MD, Gerson P, Catlin FI, Koppelman ML, Thurber S (1990). Asymptomatic congenital cytomegalovirus infection. Audiologic, neuroradiologic, and neurodevelopmental abnormalities during the first year. Am J Dis Child.

[B10] Dahle AJ, Fowler KB, Wright JD, Boppana SB, Britt WJ, Pass RF (2000). Longitudinal investigation of hearing disorders in children with congenital cytomegalovirus. J Am Acad Audiol.

[B11] Anderson KS, Amos CS, Boppana S, Pass R (1996). Ocular abnormalities in congenital cytomegalovirus infection. J Am Optom Asspc.

[B12] Temple RO, Pass RF, Boll TJ (2000). Neuropsychological functioning in patients with asymptomatic congenital cytomegalovirus infection. J Devlop Behav Pediatr.

[B13] Conboy TJ, Pass RF, Stagno S, Alford CA, Myers GJ, Britt WJ, McCollister FP, Summers MN, McFarland CE, Boll TJ (1987). Early clinical manifestations and intellectual outcome in children with symptomatic congenital cytomegalovirus infection. J Pediatr.

[B14] Saigal S, Lunyk O, Larke RP, Chernesky MA (1982). The outcome in children with congenital cytomegalovirus infection. A longitudinal follow-up study. Am J Dis Child.

[B15] Kashden J, Frison S, Fowler K, Pass RF, Boll TJ (1998). Intellectual assessment of children with asymptomatic congenital cytomegalovirus infection. J Dev Behav Pediatr.

[B16] Boppana S, Amos C, Britt W, Stagno S, Alford C, Pass R (1994). Late onset and reactivation of chorioretinitis in children with congenital cytomegalovirus infection. Pediatr Infect Dis J.

[B17] Fowler KB, Dahle AJ, Boppana SB, Pass RF (1999). Newborn hearing screening: will children with hearing loss caused by congenital cytomegalovirus infection be missed?. J Pediatr.

[B18] Stagno S, Remington JS, Klein JO (1990). Cytomegalovirus. Infectious diseases of the fetus and newborn infant.

[B19] Boppana S, Pass RF, Britt WJ, Stagno S, Alford CA (1992). Symptomatic congenital cytomegalovirus infection: neonatal morbidity and mortality. Pediatr Infect Dis J.

[B20] Bale JF, Blackman JA, Sato Y (1990). Outcome in children with symptomatic congenital cytomegalovirus infection. J Child Neurol.

[B21] Pass RF, Stagno S, Myers GJ, Alford CA (1980). Outcome of symptomatic congenital cytomegalovirus infection: results of long-term longitudinal follow-up. Pediatrics.

[B22] Williamson WD, Demmler GJ, Percy AK, Catlin FI (1992). Progressive hearing loss in infants with asymptomatic congenital cytomegalovirus infection. Pediatrics.

[B23] Williamson W, Desmond M, LeFevers N (1982). Symptomatic congenital cytomegalovirus. Disorders of language, learning and hearing. Am J Dis Child.

[B24] Rivera LB, Boppana SB, Fowler KB, Britt WJ, Stagno S, Pass RF (2002). Predictors of hearing loss in children with symptomatic congenital cytomegalovirus infection. Pediatrics.

[B25] Ross SA, Fowler KB, Ashrith G, Stagno S, Britt WJ, Pass RF, Boppana SB (2006). Hearing loss in children with congenital cytomegalovirus infection born to mothers with preexisting immunity. J Pediatr.

[B26] Fowler KB, Boppana SB (2006). Congenital cytomegalovirus (CMV) infection and hearing deficit. J Clin Virol.

[B27] Hicks T, Fowler KB, Richardson M, Dahle A, Adams L, Pass RF (1993). Congenital cytomegalovirus infection and neonatal auditory screening. J Pediatr.

[B28] Lagasse N, Dhooge I, Govaert P (2000). Congenital CMV-infection and hearing loss. Acta Oto-Rhino-Laryngologica Belgica.

[B29] Australian Institute of Health and Welfare (AIHW) (2001). Australian hospital statistics 1993–2001.

[B30] ABS (2002). Australian Bureau of Statistics: Australian Demographic Statistics. http://www.abs.gov.au/.

[B31] Porath A, McNutt RA, Smiley LM, Weigle KA (1990). Effectiveness and cost benefit of a proposed live cytomegalovirus vaccine in the prevention of congenital disease. Rev Infect Dis.

[B32] Schleiss M (2005). Progress in cytomegalovirus vaccine development. Herpes.

[B33] Hatherley L (1985). The incidence of cytomegalic inclusion disease (CID) in an obstetric teaching hospital, 1975–1984. Aust N Z J Obstet Gynaecol.

[B34] Gaytant MA, Steegers EAP, Semmekrot BA, Merkus HMMW, Galama JMD (2002). Congenital cytomegalovirus infection: review of the epidemiology and outcome. Obstet Gynecol Surv.

[B35] Sfameni SF, Skurrie IJ, Gilbert GL (1986). Antenatal screening for congenital infection with rubella, cytomegalovirus and toxoplasma. Aust N Z J Obstet Gynaecol.

[B36] Munro SC, Trincado D, Hall B, Rawlinson WD (2005). Symptomatic infant characteristics of congenital cytomegalovirus disease in Australia. J Pediatr Child Health.

[B37] Cytomegalovirus. http://www.inopsu.com/index.html.

[B38] Lynn R, Kirkbride H, Preece M, Rahi J (2003). British Paediatric Surveillance Unit- Annual Report 2002–2003.

[B39] Vaudry W, Canadian Paediatric Surveillance Program (CPSP) (2007). Congenital Cytomegalovirus infection. 2007 Results Edmonton.

[B40] Griffiths PD, Mclean A, Emery VC (2000). Encouraging prospects for immunisation against primary cytomegalovirus infection. Vaccine.

[B41] Adler S, Starr S, Plotkin S (1995). Immunity induced by primary human cytomegalovirus infection protects against secondary infection among women of childbearing age. J Infect Dis.

[B42] Stagno S, Reynolds DW, Huang ES, Thames SD, Smith RJ, Alford CA (1977). Congenital cytomegalovirus infection. N Engl J Med.

